# Exercise Classification in Resistance Training: A Systematic Review of Technological Approaches

**DOI:** 10.1007/s40279-025-02281-8

**Published:** 2025-07-31

**Authors:** Tayah R. Brennan, Jonathon Weakley, Rich D. Johnston, Mark W. Creaby

**Affiliations:** 1 School of Behavioural and Health Sciences, Australian Catholic University, Brisbane, 1100 Nudgee Road, Banyo, QLD 4014 Australia; 2 Sports Performance, Recovery, Injury, and New Technologies (SPRINT) Research Centre, Australian Catholic University, Brisbane, Australia; 3 Carnegie Applied Rugby Research Centre, Institute for Sport, Physical Activity, and Leisure, Leeds Beckett University, Leeds, UK

## Abstract

**Background:**

Modern sensor technology allows for objective tracking of resistance training exercises, yet the accuracy with which these technological approaches can classify which exercise is being completed is mixed. With commercially available technology commonly claiming the ability to characterise resistance training variables (e.g. exercise type and volume), synthesis of the current evidence base is warranted.

**Objectives:**

The aims of this systematic review were to (1) summarise the methodologies which have been used to achieve exercise prediction in resistance training and (2) compare the predictive performance of technologies and predictive models.

**Methods:**

A systematic search of four databases was performed. Included studies were: development and/or validation studies; concerned with the measurement of kinetics and/or kinematics of resistance training exercises; and used statistical prediction modelling for exercise classification. The Critical Appraisal and Data Extraction for Systematic Reviews of Prediction Modelling Studies (CHARMS) tool was used for data extraction, and the Prediction Model Risk of Bias Assessment Tool (PROBAST) tool was used to assess risk of bias and applicability. A total of 44 studies were included (2 validation; 42 development and validation studies).

**Results:**

Various technologies have been evaluated, namely: inertial measurement units, accelerometers, electromyography, electrocardiography, two-dimensional (2D) cameras, force-sensitive resistors, stretch sensors, capacitive proximity sensors, cellular signal receivers, active sonar systems, passive radio frequency identification tags, rotary encoders, and load cells. Inertial measurement units appear to be the most accurate technology available and, when worn on the wrist of the athlete, offer excellent accuracy, even for lower body exercises. Other measurement technology worn by the athlete, such as electromyography and smart materials, also offer very good accuracy. Externally placed devices, whilst offering excellent accuracy, have practical limitations that may compromise their feasibility. Of note, the exercises included the classification problem, and specifically, how similar the exercises were had a significant impact on accuracy.

**Conclusions:**

Standardising the classification problem is strongly recommended as it will likely facilitate a clearer understanding of the best approach and inform consumers and future research into this area. Furthermore, ensuring technologies are robust to the prediction of a large range of exercises with similar movement patterns remains a priority and potential barrier to feasibility. Overall, accurate exercise classification is possible with sensor-based technology, although end-user availability of such technology is limited. It is strongly advised that users be cautious of consumer-level technology, because few are scientifically validated.

**Supplementary Information:**

The online version contains supplementary material available at 10.1007/s40279-025-02281-8.

## Key Points

**Table:** 

Wrist-worn inertial measurement units appear to be the most accurate technology, offering excellent accuracy, even for classifying lower body exercises.
End-user availability of technology for accurate classification of resistance training exercise is limited. It is strongly advised that users be cautious of consumer-level technology, because few are scientifically validated.
For researchers, a standardised approach to the exercise classification problem is strongly recommended; we propose a three-phased method to facilitate rigorous and comparable outcomes of technological solutions to the classification and tracking of resistance training exercises.

## Introduction

Monitoring training is important for improving adherence to exercise programs, increasing motivation to exercise, minimising the risk of injury and improving physical adaptations [1]. Technologies designed to monitor exercise performance are widely available, yet these have largely focused on endurance exercise, such as running and cycling. Resistance training is commonly used to improve musculoskeletal health and is integral to many athlete training programs. Typically, methods of quantifying loads in resistance training involve manual recording of data and calculations of total volume and intensity [1]. These methods can be misleading, particularly when differences in training structure, strategies or performance (e.g. range of motion) are present, as physiological and adaptive responses will vastly differ [2]. With the proliferation of sensor-based technology, a range of approaches have emerged that have the potential to advance the quantification and analysis of resistance training. Many of these technologies are shown to be valid and reliable tools for the monitoring of training load during resistance training [3, 4] and are commonly used to support decision-making regarding exercise programming [5]. However, most of these technologies are limited to use with free weights, require users to manually select the exercises being performed and may also require users to manually start and stop recording of each exercise. As a result, users may forget to change or stop the exercise, particularly when different set structures, such as supersets, tri-sets and circuits are performed [2], making these devices hard to use on an everyday basis by exercisers. Accurate, objective and automated approaches to quantify resistance training loads and performance would represent a significant tool in strength and conditioning coaches’ arsenal to monitor training adherence, performance and inform decisions regarding future training load.

The development of a usable system that can identify the exercise (and other performance metrics) being performed involves a process known as prediction modelling. Broadly, prediction modelling employs algorithms to build and train models that predict outcomes on the basis of data features [6]. Whilst there is a common process used in prediction model studies, different methodologies have been employed across literature, and their predictive performance varies [7, 8]. Typically, researchers identify an outcome (e.g. the exercises they aim to predict) relevant to a population in which the model is intended to be used [6]. For instance, exercises linked to performance outcomes or exercises that are commonly performed by certain populations may be selected. This means that the number of exercises and the exercise modality (e.g. free-weight, bodyweight or machine-resisted) varies across literature [9, 10]. Furthermore, theoretically, a more complex classification problem (e.g. an increased number of potential outcomes) will result in worse model performance, potentially biasing the interpretation of model performance.

A key component of prediction modelling is the gathering of the data which will be fed into statistical algorithms. Inertial measurement units (IMUs), stand-alone accelerometers, vision-based sensors and a range of other technologies, including custom-built devices, have been used in literature for the measurement of biomechanical variables during exercise. As the construct(s) each approach measures, and their measurement properties, will differ, it is likely that their predictive performance will also differ. Thus, an understanding of which technology, physical location(s) and required number of sensors performs the best for which exercises is warranted. This will guide technology choice for athletes and coaches, as well as inform which measurement tools merit further research and development.

The most common process used to produce a model for predicting human activities involves machine learning algorithms [11]. Numerous approaches have been employed; namely, neural networks, discriminant analysis and tree-based algorithms, each with varying performance. These algorithms work in different ways to recognise patterns or discriminating features in the input data to classify the data into a desired output [12–14]. Given that the algorithm used will likely affect performance, it is important to identify which performs the best for addressing exercise classification problems.

With the proliferation of research addressing the resistance training exercise classification problem in recent years, a synthesis of the available literature will expedite the development of promising approaches and inform practitioners of current best solutions. These improvements will support effective load monitoring in the gym and potentially lead to enhanced training prescription. Therefore, the aim of this review is to establish the current level of evidence for the technological approaches to classifying resistance training exercises: specifically, to (1) summarise the methodologies which have been used to achieve exercise prediction in resistance training and (2) compare the predictive performance of technologies and models.

## Methods

A systematic review was carried out in accordance with the Preferred Reporting Items for Systematic Reviews and Meta-Analyses (PRISMA) guidelines [15, Supplementary Material S1]. The study protocol was pre-registered on 21 May 2024 (PROSPERO, 2024: CRD42024535133).

### Study Eligibility Criteria

To be eligible for inclusion, studies were required to (1) have human participants; (2) be development and/or validation studies; (3) assess any combination of resistance training exercise; (4) be concerned with the measurement of the kinetics and/or kinematics of resistance training using technology; and (5) have used statistical prediction modelling for exercise classification. Studies were excluded if they were (1) books, book chapters, letters, editorials, reviews, or conference papers; (2) studies with no exercise prediction and/or classification; or (3) studies that predicted and/or classified the incidence of a single event (i.e. did not differentiate between various exercises).

### Information Sources and Search Strategy

The academic databases SPORTDiscus, Web of Science, PubMed and Scopus were systematically searched from the earliest record to 19 May 2024 and updated on 25 October 2024. Using titles, abstracts, keywords and Boolean operators, peer-reviewed original research studies that investigated the predictive ability of technology in resistance training were identified. In addition, reference lists of eligible studies were manually searched for any potential articles that were not retrieved in the initial search. The following search string was used, with medical subheadings (MeSH) incorporated in the PubMed search:

(((weightlift* OR ‘weight lift*’ OR ‘resistance train*’ OR ‘resistance exercis*’ OR ‘weight train*’ OR powerlift* OR ‘power lift*’ OR ‘strength train*’ OR ‘strength exercis*’ OR workout* OR CrossFit OR ‘free weight*’ OR ‘gym exercise machine*’) OR (Resistance Training[MeSH Terms])) AND ((‘inertial sensor*’ OR microsensor* OR sensor* OR wearable* OR ‘inertial measurement unit’ OR IMU OR accelerom* OR gyroscope OR gonio* OR ‘wireless body sensor network’ OR WBSN OR ‘linear position transducer’ OR ‘linear velocity transducer’ OR ‘linear encoder’ OR ‘motion analysis’ OR ‘motion capture’ OR ‘laser optic’ OR smartwatch* OR ‘Push band’ OR ‘fine-grain*’) OR (Wearable Electronic Devices[MeSH Terms]))) AND ((predict* OR detect* OR recogni* OR classif* OR ‘machine learning’ OR ‘deep learning’ OR ‘neural network*’ OR regression OR PCA OR KNN OR SVM OR CNN OR ‘decision tree’ OR ‘random forest’) OR (Biomechanical Phenomena[MeSH Terms]))

### Study Selection

All search results were extracted and imported into a reference manager (EndNote 21, Clarivate, Philadelphia, PA, USA). Duplicates were removed. Two reviewers (T.B. and M.C.) independently screened the titles, abstracts and full texts of articles against the eligibility criteria. Any disagreements were resolved by a consensus discussion between T.B. and M.C. Endnote and Excel (Microsoft, Washington, USA) were used for the screening and recording of all decisions.

### Data Items and Collection

A modified version of the Critical Appraisal and Data Extraction for Systematic Reviews of Prediction Modelling Studies (CHARMS) tool by Fernandez-Felix et al. [16], adapted from Moons et al. [17], was used to extract data from the included studies. To account for the nature of our research question and how it differs from traditional prognostic and/or diagnostic studies for which the CHARMS tool is designed, modifications were made in several sections (study information, candidate predictors, sample size and model performance), and new sections (technology and system availability) were added (Supplementary Material S2). The authors of this review chose to select only the model with the best performance from each of the included studies; however, when different data (i.e. study population, outcome, or measurement tools) were reported in the same paper for multiple models, each of these models was included. When several classification problems were assessed with the same model, the classification problem with the most exercises was included. When subject-dependent and subject-independent models were reported in the studies, data were extracted for the subject-independent model. If studies developed and evaluated models for other performance metrics, data for these models were also extracted. Two reviewers (T.B. and M.C.) independently extracted data from each of the included studies, and extracted data were checked by a third reviewer (R.J.). Any disagreements between judgments of extracted data were resolved by consensus discussion. When possible, to obtain any desired but unreported data, data were calculated from other information reported.

### Risk of Bias and Applicability Assessment

To assess the risk of bias and applicability of the included studies, a modified version of the Prediction Model Risk of Bias Assessment Tool (PROBAST) tool by Fernandez-Felix et al. [16], adapted from Moons et al. [18], was used. Additional signalling questions were needed so that this assessment tool was applicable to our research question (Supplementary Material S2). As per PROBAST, study risk of bias and applicability were scored unclear (no information), low (yes, or probably yes), or high (no, or probably no) in each domain. An overall rating was reached on the basis of the classification in each domain; that is, if all domains had low risk and low concern for applicability, then overall judgement was low, if at least one domain had high risk and high concern for applicability, then overall judgment was high, and if unclear risk of bias and applicability was noted in at least one domain and all other domains had low risk and low concern, the overall judgement was unclear [16, 18]. The number of events (i.e. the number of participants with the outcome) per variable (EPV) was used to assess sample sizes of the included studies, whereby studies with a sample size with an EPV of less than ten were considered at risk of overfitting [17]. Risk of bias was assessed independently by two reviewers (T.B. and M.C.) for each of the included studies. Any disagreements were resolved by consensus discussion.

### Synthesis Methods

Data pertaining to the model development methodology, predictor variables, model validation and model performance measures were tabulated according to the technologies utilised in the included studies. To explore possible causes of heterogeneity among the study results, sub-group analysis for each of the included technologies was performed. Of note, we indicate data acquisitions processes, the adopted classification algorithms and practices for model performance reporting. To prepare the data for presentation and synthesis, some data conversions were required so that measures were consistent across the studies (e.g. height data reported in metres was converted to centimetres).

## Results

### Study Selection

**Fig. 1:**
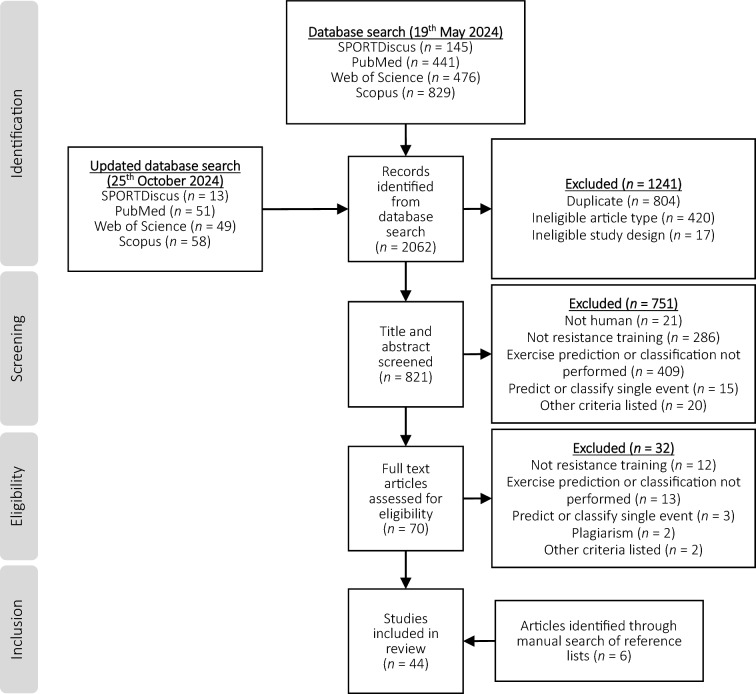
Flow diagram of screening and selection process

Of the 2062 total studies retrieved from the systematic search, 38 met the criteria for inclusion in the review. With the addition of 6 eligible studies identified via manual searching of reference lists, a final 44 studies were included (Fig. 1). Two studies appeared to meet the criteria for inclusion in the systematic review but were excluded after being identified as plagiarising the results (and other aspects) of included studies. Two studies reported exercise classification for two different technologies and a combination of the technologies [10, 55], and another study reported exercise classification models in two separate samples [19], resulting in 49 prediction models in total.

### Study Characteristics

**Fig. 2:**
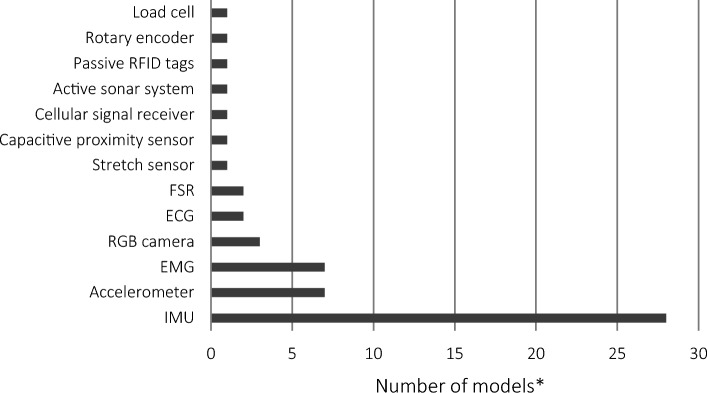
Overview of the technologies utilised in the reviewed models. ^*^The number of models that used each of the technologies, including if used in combination with other technologies. *ECG* electrocardiogram, *EMG* electromyography, *FSR* force-sensitive resistor, *IMU* inertial measurement unit, *RFID* radio frequency identification, *RGB* red, green, blue

Of the 44 included studies, two were validation studies and the remaining 42 were both development and validation studies (Supplementary Material S2). The number of participants enrolled in the studies ranged from 1 to 159, although only seven of the reviewed studies collected data from more than 30 participants. Studies often included male and female participants aged between 18 and 65 years, with training experience ranging from no experience to highly trained individuals. Most studies did not report training experience or gave a broad description of trained and untrained; the specific descriptors of training experience for each study are provided in Supplementary Material S2. Of the 49 models, most used IMUs (24/49), followed by accelerometers (6/49), then a range of other technologies (11/49) (Fig. 2). In addition, some models used a combination of technologies in their methodology (8/49).

### Risk of Bias and Applicability Assessment

**Fig. 3:**
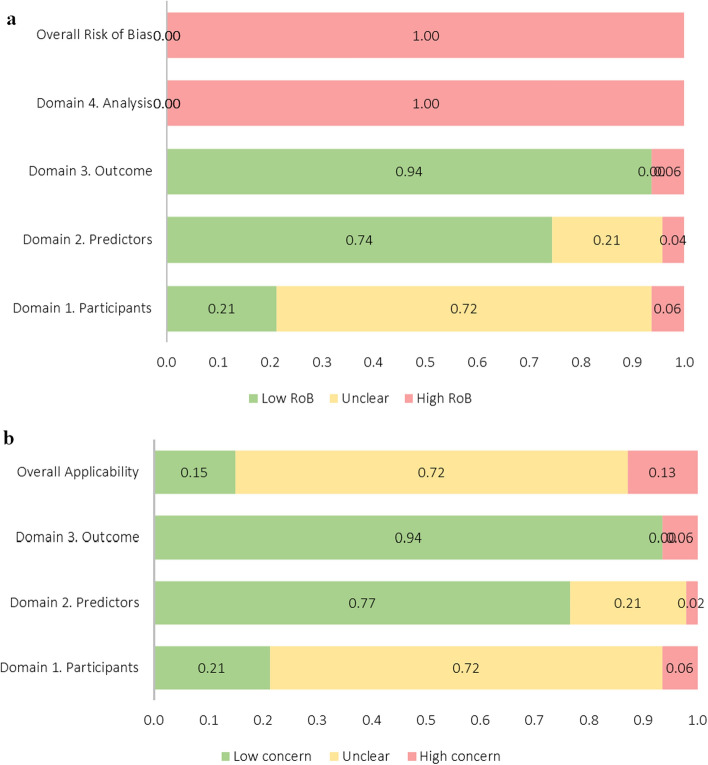
Summary of the overall and domain risk of bias (**a**) and applicability (**b**) assessments. Domain one (participants) covers potential sources of bias and concerns related to the applicability of the selected participants and/or sources of data; domain 2 (predictors) covers potential sources of bias and concerns related to the applicability of the definition and measurement of predictors; domain 3 (outcome) covers potential sources of bias and concerns related to the applicability of the definition and measurement of the outcome classified by the model; and domain 4 (analysis) covers potential sources of bias related to the analysis of the outcome [16]. *RoB* risk of bias

All the included models presented with a high risk of bias and most presented with an unclear concern regarding the applicability to the review (*n* = 36) (Fig. 3; Supplementary Material S2). Briefly, a lack of transparent reporting was evident in many of the reviewed models. Factors contributing to risk of bias included inadequate reporting of sample characteristics (21/49), failure to report sample eligibility criteria (39/49), inadequate reporting of missing data (41/49), failure to report predictors (12/49), inappropriate evaluation of model performance measures (i.e. no evaluation of discrimination and calibration) (48/49), and no internal validation or only split-sample internal validation performed (11/49). Additionally, 24 models were determined to have a small sample size (EPV < 10), and sample size could not be assessed for 25 models owing to inadequate reporting.

### Inertial Measurement Units (IMUs)

#### Specifications

**Fig. 4:**
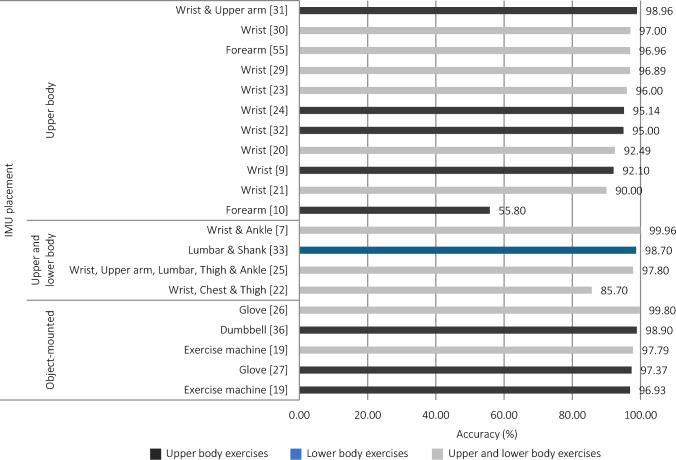
Overview of reported accuracy measures for inertial measurement unit (IMU)-based models according to the placement of devices and exercises included in the classification problem

Inertial measurement units were the most frequently used technology for classifying resistance training exercises (24/49). Studies that used IMUs opted for stand-alone IMUs or built-in sensors, such as those found in modern smartwatches. Sampling rates in the reviewed studies ranged from 10 to 512 Hz. Smaller sampling rates were seen in studies where a good trade-off between accuracy and energy optimisation was a priority (e.g. IMUs sampled at 40 Hz [34]). Collectively, sampling rate did not appear to be related to model performance, but it is worth noting that studies with models that performed the best captured data between 100 and 128 Hz. Of the 24 models that utilised IMU data, individual sensor data consisted of accelerometer data only (*n* = 6), accelerometer and gyroscope data (*n* = 8), accelerometer and magnetometer data (*n* = 2), and accelerometer, gyroscope and magnetometer data (*n* = 8) (Table 1). Broadly speaking, models that used data from all three sensors appeared to perform best. The number of IMUs that were utilised in these studies ranged between one and five, and were affixed to segments of the upper body (*n* = 15), upper and lower body (*n* = 4), or were mounted on an object that was used during exercise (e.g. mounted on a dumbbell [36]) (*n* = 5). The number of IMUs used appeared to have no effect on model performance. However, some studies showed that utilising sensors on both the upper and lower body (e.g. wrist and ankle [7]) performed better than when devices were worn only on the upper body (Fig. 4). That said, a single IMU on the wrist was the most common location and appeared to offer excellent accuracy, even when lower body exercises were included in the classification problem.

**Table 1:** Summary of studies that investigated inertial measurement units (IMUs) for the prediction of exercise during resistance training (*n* = 24)

IMU sensor	Study	Exercise	Predictors	Prediction model algorithm	Validation method	Performance variables	Findings
Accelerometer (*n* = 6)	Koo et al. [20]	Bench press, incline bench press, shoulder press, overhead triceps extension, dumbbell kickback, front raise, lat pulldown, straight arm pulldown, deadlift, dumbbell row, one-arm dumbbell row, EZ-bar curls, machine preacher curl, lateral raise	Time-series *z*-axis acceleration data	Combined methods; decision tree (DT) and convolution neural network (CNN)	Train and test only	Accuracy	ACC: 92.49
	Lim et al. [21]	Squat, push-up, lunge, jumping jack, bench press, good morning, deadlift, push press, back squat, arm curl, military press, bent-over row, burpee, leg-raised crunch, lateral raise	NI	Dynamic time warping (DTW)	Other	Accuracy, F1-score, receiver operating characteristic curve, area under the receiver operating characteristic curve, mean detection rate	ACC: 89.2; F1: 89.4; AUC: 0.936; detection rate: 93.09
	Džaja et al. [22]	Front raise, lateral raise, dumbbell shrug, dumbbell curl, bent-over row, push-up, step ups, box squat, heel touch	Resultant acceleration signal magnitude	Dynamic time warping (DTW)	Train and test only	Accuracy	ACC: 85.7
	Sun et al. [36]	Single-arm row, single-arm triceps extension, dumbbell biceps curl, lateral raise, dumbbell bench press, dumbbell chest fly	Mean, standard deviation, maximum, minimum, mean absolute deviation, interquartile distance, range, skewness, absolute mean value, crest factor, impulse factor, form factor	Neural network (NN)	Train and test with validation split	Accuracy	ACC: 98.9
	Jeong et al. [23]	Pull-up, barbell row, bench press, triceps dips, squat, deadlift, military press	Time series data	One-dimensional convolution neural network (CNN)	Train and test with validation split	Validation accuracy, test accuracy	Validation ACC: 99; test ACC: 96
	Koskimäki and Siirtola [10]	Close-grip bench press, skull crusher, triceps pushdown, triceps dips, overhead triceps extension, triceps dumbbell kickback, spider curl, dumbbell alternating biceps curl, incline hammer curl, concentration curl, cable curl, hammer curl, upright row, lateral raise, front raise, shoulder press, car drivers, lying rear delt raise, bench press, incline dumbbell fly, incline dumbbell press, dumbbell fly, push-up, leverage chest press, seated cable row, one-arm dumbbell row, behind-neck lat pulldown, barbell row, reverse grip, reverse-grip barbell row, lat pulldown	Mean, standard deviation, minimum, maximum, median, percentiles, zero and mean crossing, Fast Fourier Transform sums, squared sums using all channels, sums of wavelet decompositions using different bookkeeping vectors, autocorrelation, cross-correlation	Unclear; linear discriminant analysis (LDA) and/or quadratic discriminant analysis (QDA)	Train and test with leave-one-out cross validation	Accuracy	ACC: 55.8
Accelerometer, gyroscope (*n* = 8)	Preatoni et al.[25]	Clean and jerk, box jump, kettlebell swing, burpee	Mean value, standard deviation, root mean square, mean absolute deviation, max value, min value, kurtosis, skewness, quartile (25th, 50th, 75th), power, higher frequency, lower frequency, median frequency, mean frequency, spectral entropy	Support vector machines (SVM)	Train and test with fivefold cross validation and leave-one-out cross validation	Accuracy	ACC: 97.8
	Ahn et al. [24]	Long-pull, high-pull, shoulder press, chest press, bench press	Minimum, maximum, minimum–maximum difference, mean, standard deviation, root mean square	Multilayer perceptron	Train and test with tenfold cross validation	Accuracy	ACC: 95.14
	Burns et al. [9]	Pendulum, shoulder abduction, shoulder forward elevation, shoulder internal rotation, shoulder external rotation, trapezius extension, upright row	Mean, variance, standard deviation, maximum, minimum, skewness, kurtosis, mean crossings, mean spectral energy, 4-bin histogram	Convolutional recurrent neural network (CRNN)	Train and test with temporal and subject stratified fivefold cross validation	Accuracy	ACC: 99.996 (temporal), 92.1 (subject)
	Zou et al. [27]	Dumbbell curl, hammer curl, shoulder external rotation, chest press, reverse-grip pulldown, face pulls, dumbbell fly, triceps extension, Arnold lift, dumbbell lateral curl, bent-over row, bench press, pec dec fly, incline dumbbell fly, cable curl	NI	Dynamic time warping (DTW)	Other	Accuracy, computational (time) delay	ACC: 97.37; delay: 174.66 ms
	Shen et al. [28]	Machine biceps curl, triceps extension, lat pulldown, chest press, shoulder press, seated row, pec fly, rear delt fly, triceps dip, ab crunch, dumbbell biceps curl, overhead triceps extension, dumbbell single arm row, lateral raise, bench press	Mean, standard deviation, minimum, maximum, range	Combined methods; conditional random fields (CRF), decision tree hidden Markov model (DT-HMM), and Gaussian support vector machines (SVM)	Train and test with tenfold cross validation	Precision, sensitivity, F1-score	PPV: 89.71; TPR: 89.53; F1-score: 89.48
	Prabhu et al. [29]	Biceps curl, front raise, lateral raise, triceps extension, pec dec, trunk twist, squats, lunge, leg lateral raise, standing bicycle crunches	Minimum, maximum, mean, standard deviation, root mean square, entropy, energy from Fast Fourier Transform coefficient, Pearson correlation coefficient	Convolution neural network (CNN)	Train and test with validation split	Accuracy, misclass	ACC: 96.89; Misclass: 3.11
	Crema et al. [30]	Squats, deadlifts, rowing, bench press, shoulder press, biceps curl, French press, lateral raise, bent-over lateral raise	Amplitude frequency distribution within five classes, root mean square, mean, standard deviation, power bands, autocorrelation maximum value, prominent peaks, weak peaks, value of the first autocorrelation peak after a zero-crossing, kurtosis, interquartile range	Combined methods; principal component analysis (PCA) and linear discriminant analysis (LDA)	Train and test with leave-one-out cross validation	Accuracy, precision, sensitivity	ACC: 97; PPV: 90; TPR: 93
	Mekruksavanich and Jitpattanakul [55]	Seated cable rows, one-arm dumbbell row, wide-grip pulldown behind the neck, bent-over barbell row, reverse-grip bent-over row, wide-grip front pulldown, bench press, inclined dumbbell flyers, inclined dumbbell press, dumbbell flyers, push-ups, leveraged chest press, closed-grip barbell bench press, bar skull crusher, triceps pushdown, bench dip/dip, overhead triceps extension, triceps dumbbell kickback, spider curl, dumbbell alternate biceps curl, inclined hammer curl, concentration curl, cable curl, hammer curl, upright barbell row, side lateral raise, front dumbbell raise, seated dumbbell shoulder press, car drivers, lying rear-delt raise	NI	Combined methods; convolution neural network, residual connections, and bidirectional gated recurrent units (CNN-ResBiGRU)	Train and test with fivefold cross validation	Accuracy, F1-score, loss	ACC: 96.96; F1: 91.78; Loss: 0.14
Accelerometer, magnetometer (*n* = 2)	Radhakrishnan et al.(Study1_univ) [19]	Triceps extension, lat pulldown, biceps curl, cable wood chop, bent-over raise, seated row, chest press, face pulls, upright row, wrist curl	Mean, max, min, range, variance, spectral entropy, spectral energy, mean crossing rate, covariance, correlation, repetition time, repetition height, repetition velocity mean, repetition velocity standard deviation	Random forest (RF)	Train and test with tenfold cross validation	Accuracy, precision, sensitivity	ACC: 96.93; PPV: 96.2; TPR: 96.9
	Radhakrishnan et al. (Study2_comm) [19]	Leg curls, leg press, triceps pushdown, biceps curl, chest press, shoulder press	Mean, max, min, range, variance, spectral entropy, spectral energy, mean crossing rate, covariance, correlation, repetition time, repetition height, repetition velocity mean, repetition velocity standard deviation	Random forest (RF)	Train and test with tenfold cross validation	Accuracy, precision, sensitivity	ACC: 97.79; PPV: 97.8; TPR: 98.2
Accelerometer, gyroscope, magnetometer (*n* = 8)	Wu et al. [31]	Biceps curl, lateral raise, shoulder press	3-axis accelerations and angular velocities	Long short-term memory (LSTM)	Train and test only	Accuracy	ACC: 98.96
	Guo et al. [32]	Barbell bench press, dumbbell bench press, rear delt fly, lat pulldown, machine chest press, cable cross-over, cable biceps curl, dumbbell biceps curl, dumbbell row, dumbbell raise, rowing, running	Skewness, kurtosis, standard deviation, variance, median, range, trimean, mean, most frequently appear in the array	Support vector machines (SVM)	Train and test with tenfold cross validation	Accuracy	ACC: 95
	O'Reilly et al. [33]	Squats, deadlifts, lunges, single-leg squats, tuck jump	Mean, root mean square, standard deviation, kurtosis, median, skewness, range, variance, max, index of max, min, index of min, energy, 25th percentile, 75th percentile, level crossing rate, fractal dimension, variance of the approximate and detailed wavelet coefficients using the Daubechies 4 mother wavelet to level 7	Random forest (RF)	Train and test with leave-one-out cross validation	Accuracy, sensitivity, specificity	ACC: 98.7; TPR: 98.66; TNR: 99.67
	Coates and Wahlström [34]	Biceps curl, lateral raise, shoulder press	Maximum, minimum, gravity height, rotation symmetry, roll height, gravity/roll turning points, mean, standard deviation, average absolute difference, minimum–maximum difference, median, median absolute deviation, interquartile range, number of values above the mean, number of local maxima, skewness, kurtosis, energy, average resultant acceleration, signal magnitude area	Gradient boosting	Train and test only	Sensitivity	TPR: 100
	Kwon et al. [35]	One-arm dumbbell row, incline dumbbell fly, incline dumbbell press, dumbbell fly, dumbbell kickback, biceps curl, incline hammer curl, concentration curl, hammer curl, lateral raise, front raise, shoulder press, rear delt raise	NI	Recurrent neural network (RNN)	Train and test only	F1-score	F1: 80.2
	Balkhi and Moallem [26]	Squat, hammer curl, lateral raise, single arm dumbbell row, straight arm pullover, seated cable row, standing triceps extension, shoulder press, bent-over raise	Mean, root mean square, maximum, minimum, standard deviation, variance, skewness, kurtosis	Random forest (RF)	Train and test with tenfold cross validation	Validation accuracy, test accuracy, precision, sensitivity, F1-score	Validation ACC: 98.89; Test ACC: 99.8; PPV: 97; TPR: 89; F1-score: 94
	Soro et al. [7]	Push-up, pull-up, burpee, kettlebell deadlift, box jump, air squat, sit-up, wall ball, kettlebell press, kettlebell thruster	Raw readings from accelerometer, gyroscope and orientation data	Convolution neural network (CNN)	Train and test with fivefold cross validation	Accuracy	ACC: 99.96
	Czekaj et al. [56]	Abdominal tenses, standing-to-plank-downward-dog-to-plank sequences, lying hip rises, side lunges, sit-to-stands, broad jumps, burpees, crunches, lunges, push-ups, squats	NI	Convolutional neural networks (CNN) with two stacked bidirectional long short-term memory (LSTM)	Train and test with threefold cross validation	No overall performance measures	

*NI* no information reported by the study, *ACC* accuracy, *PPV* positive predictive value, Precision; *TPR* true positive rate, Sensitivity; *TNR* true negative rate, Specificity; *AUC* area under the receiver operating characteristics curve

#### Data Acquisition

Data acquisition typically took place in a gym facility, although most studies did not report the study setting. In many studies, participants were given specific instructions regarding proper execution (e.g. form and range of motion [33, 34]) of the pre-selected exercises. A less common approach involved unconstrained workouts, where participants performed exercises as usual [21]. The IMU-based models tended to classify small sets of commonly performed free-weight (e.g. dumbbell biceps curl, dumbbell shoulder press), machine-resisted (e.g. lat pulldown, pec dec) and/or bodyweight (e.g. push-up, bodyweight squat) resistance training exercises, with the number of exercises ranging from 3 to 30, with only seven models including more than 10 exercises. Two models classified rehabilitation exercise [9, 56]. The number of exercises in the classification problem did not seem to influence model performance. Similarly, the exercise modality did not appear to influence model performance, as models seemed to perform better or worse regardless of whether exercises were performed using free-weights or machines (or a combination of both), or no external load. The selection of exercise appeared to determine sensor location(s), and this generally allowed for improved model performance when sensors were located on the body segment with the greatest movement during the exercise. However, this was not true for all models. For example, Wu et al. [31], Guo et al. [32] and Burns et al. [9] all positioned IMUs on the upper body and classified exercises targeting the upper body but achieved accuracies of 98.96%, 95.00% and 92.10%, respectively (Fig. 4). The most notable difference between these models is the observed discernability of the classified exercises. The exercises that were included in Wu et al. [31] had movement patterns that would be much easier to differentiate (e.g. biceps curl and shoulder press) than those included in Guo et al. [32] (e.g. barbell bench press and dumbbell bench press) and Burns et al. [9] (e.g. shoulder internal rotation and shoulder external rotation; Table 1), and this finding was consistent across many of the IMU-based models.

#### Model Development and Evaluation

**Fig. 5:**
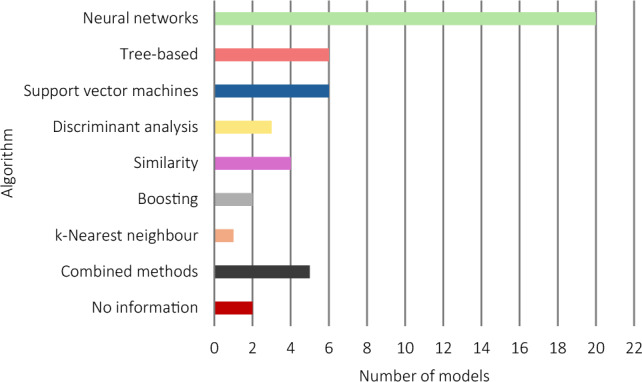
Overview of the algorithms used to produce predictive models. Bar colour corresponds to the predictive algorithm approach employed in the study: neural networks (green), tree-based (rose), support vector machines (blue), discriminant analysis (yellow), similarity (plum), boosting (grey), k-nearest neighbour (orange), combined methods (black), no information on the classification algorithm used in the study (red)

**Fig. 6:**
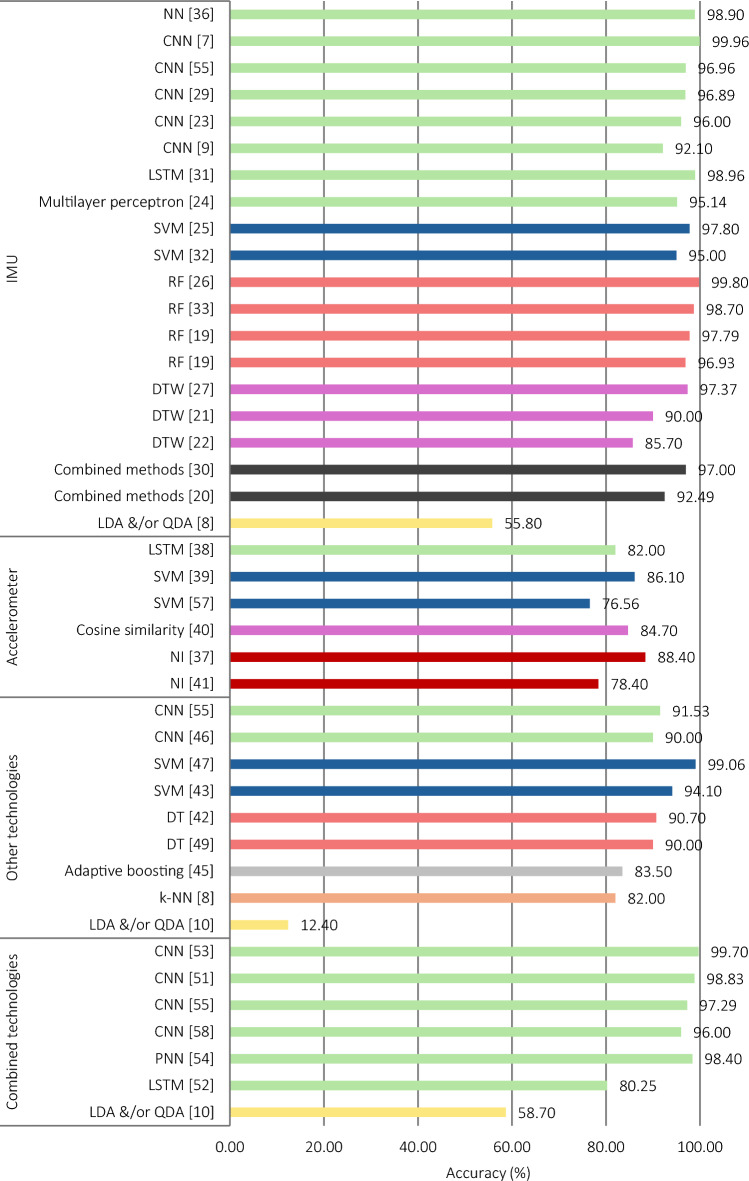
Overview of reported accuracy measures according to the technology and algorithm used to produce predictive models. Some models did not report classification accuracy. Bar colour corresponds to the predictive algorithm approach employed in the study: neural networks (green), tree-based (rose), support vector machines (blue), discriminant analysis (yellow), similarity (plum), boosting (grey), k-nearest neighbour (orange), combined methods (black), no information on the classification algorithm used in the study (red). *CNN* convolution neural networks, *DT* decision tree, *DTW* dynamic time warping, *IMU* inertial measurement unit, *k-NN* k-nearest neighbour, *LDA* linear discriminant analysis, *LSTM* long short-term memory, *NN* neural networks, *NI* no information, *PNN* probabilistic neural network, *QDA* quadratic discriminant analysis, *RF* random forest, *SVM* support vector machines

The algorithms used to build and train predictive models were diverse (Fig. 5). Convolutional neural networks (CNN) were most common for model development, followed by random forest (RF) and dynamic time warping (DTW). Significant variations were noted in the accuracies of models that were reported using the same algorithms. For instance, Burns et al. [9], Jeong et al. [23] and Soro et al. [7] each used CNN, but the accuracies of these models were found to be distinctly different (Fig. 6).

Models were typically evaluated by splitting the pre-processed dataset into training and test sets. Split-sample internal validation only, or split-sample with k-fold or leave-one-out cross-validation were most common. The predictive performance of the IMU-based models was described using classification accuracy in all but four models, and this was the only measure of model performance calculated in 11 models (Table 1). Only one of the IMU-based models’ performance was quantified using an assessment of discrimination [21]. Other models used measures derived from confusion matrices (12/24), enabling an understanding of prediction accuracy on an individual exercise level. Overall, the IMU-based models reported excellent classification accuracies, typically above 95%.

#### Other Models

In addition to addressing the exercise classification problem, nine studies reported models to predict the number of repetitions completed [7, 21, 26, 28–32, 34], five studies reported models to analyse the users’ exercise form [19, 21, 32, 34, 36] and four studies reported models for other performance metrics, such as weight detection [19, 26], one repetition maximum (1RM) prediction [51], range of motion [34] and repetition time [34]. Of those reporting repetition counting, the mean counting error varied between 0.0 and 0.59 per repetition.

### Accelerometers

#### Specifications

Some of the reviewed studies used stand-alone accelerometers (6/49). Of these, two were validation only: Oberhofer et al. [37] validated a smartwatch-based exercise analysis app for exercise classification, and Steeves et al. [41] validated a commercially available wrist-worn wearable for exercise classification. Most studies that used accelerometers developed and/or validated models on the basis of built-in accelerometers, such as in wrist or chest straps, and few used independent accelerometers. Sampling rates in the reviewed studies ranged from 30 to 100 Hz, although only four studies reported a sampling rate. On the basis of the available data, it is unlikely that the sampling rate would have a significant impact on model performance; however, this cannot be determined with any certainty. A single accelerometer affixed to the wrist or chest was used in all of the reviewed studies, except one, where five accelerometers were positioned in various locations on the upper body. The number and position of the devices did not appear to influence the performance of the models.

#### Data Acquisition

**Fig. 7:**
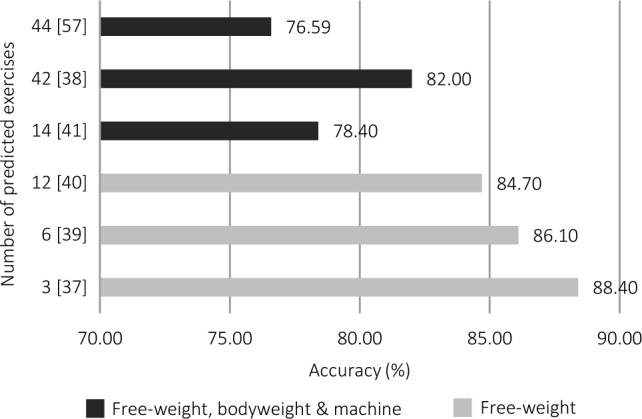
Overview of reported accuracy measures for accelerometer-based models according to the number of predicted exercises and exercise modalities

Data acquisition typically took place in a gym facility or controlled laboratory setting, although only two studies reported the study setting. In all studies, participants were given specific instructions regarding proper execution of the pre-selected exercises. The accelerometer-based models tended to classify small sets (3–14 exercises) of free-weight, machine-resisted, and/or bodyweight resistance training exercises. Two models classified a large set of 42 and 44 free-weight, machine-resisted, and bodyweight exercises, respectively [38, 57]. The number of exercises may have affected model performance with a trend towards poorer accuracy as the number of exercises increased (Fig. 7). Similarly, the models that predicted free-weight exercises appeared to perform better compared with the models that predicted a combination of modalities. When machine-resisted exercises that involve little or no movement in the upper body were included in Hussain et al. [38] (e.g. leg press and leg extension), the model appeared to perform worse than most of the other studies (Table 2).

**Table 2:** Summary of studies that investigated accelerometers for prediction of exercise during resistance training (*n* = 6)

Study	Exercise	Predictors	Prediction model algorithm	Validation method	Performance variables	Findings
Oberhofer et al. [37]	Barbell deadlift, barbell bench press, barbell back squat	NI	NI. Pre-developed app	External (completely independent)	Accuracy	ACC: 88.4
Hussain et al. [38]	Butterfly, cable crossover, chest press, decline press, dumbbell fly, incline press, push-ups, barbell biceps curl, triceps dips, dumbbell biceps curl, dumbbell extension, preacher curl, cable extension, high pulley curl, 4 × 4 dumbbell, front raise, back shoulder press, upright row, front shoulder press, lateral raise, shrugs, pull-ups, front pulldown, lower pulley, vertical traction, back pulldown, one-arm dumbbell row, T-bar row, squats, leg press, leg extension, leg curl, abductor, adductor, lunge, leg raises, crunches, cross bicycles, plank, mountain climber, leg scissor, boat	Tri-axial accelerometer time-series data	Long short-term memory (LSTM)	Train and test only	Accuracy, loss, precision, sensitivity, F1-score	ACC: 82; loss: 0.61; PPV: 81.9; TPR: 81.3; F1: 81.3
Pernek et al. [39]	Biceps curl, triceps extension, front raise, lateral raise, dumbbell row, shoulder press	Minimum, maximum, range, arithmetic mean, standard deviation, root mean square, correlation	Support vector machines (SVM)	Train and test with leave-one-out cross validation	Accuracy	ACC: 86.1
Conger et al. [40]	Bench press, shoulder press, biceps curls, upright rows, lateral raises, overhead triceps extensions, kneeling triceps kickbacks, standing bent-over rows, kneeling bent-over rows, squats, forward walking lunges, calf raises	NI	Cosine similarity	Train and test only	Accuracy, consistency (Cohen’s kappa)	ACC: 87.9; kappa statistic: 0.833 (*p* < 0.001)
Steeves et al. [41]	Dumbbell bench press, lat pulldown, goblet squat, dumbbell Arnold press, biceps curl, crunch, push-up, dumbbell upright row, lunge, lateral raise, triceps extension, deadlift, dumbbell bent-over row, calf raise	NI	NI. Commercially available device	External (completely independent)	Accuracy, repeated measures ANOVA, detection error	ACC: 78.4; ANOVA: significant difference from 100% accuracy (*p* < 0.5); error: 2%
Hussain et al. [57]	Butterfly, cable crossover, chest press, decline press, dumbbell fly, incline press, push-ups, barbell biceps curl, triceps dips, dumbbell biceps curl, dumbbell extension, preacher curl, cable extension, triceps kickback, high pulley curl, 4 × 4 dumbbell, front raise, back shoulder press, upright row, front shoulder press, lateral raise, shrugs, pull-ups, front pulldown, lower pulley, vertical traction, back pulldown, one-arm dumbbell row, T-bar row, squats, leg press, leg extension, leg curl, abductor, adductor, lunge, leg raises, crunches, cross bicycles, plank, mountain climber, leg scissor, boat, decline twister ball	Mean, standard deviation, average absolute deviation, minimum value, maximum value, difference of max and min values, median, median absolute deviation, interquartile range, number of values above mean, number of peaks, skewness, kurtosis, energy, average resultant acceleration, signal magnitude area, index od max value in time domain, index of min value in time domain, absolute difference between index of max and min in time domain, index of max in frequency domain, index of min in frequency domain, absolute difference between index of max and min in frequency domain	Support vector machine (SVM)	Grid search cross validation	Accuracy	ACC: 76.59

*NI* no information reported by the study, *ACC* accuracy, *PPV* positive predictive value, Precision; *TPR* true positive rate, Sensitivity; *ANOVA* analysis of variance

#### Model Development and Evaluation

The algorithms used to build and train predictive models differed across the studies (Fig. 5). Two studies did not report the algorithm used to produce predictions [37, 41]. Of the studies that developed models, cosine similarity appeared to be the top performing algorithm for classifying resistance training exercise with accelerometers (Fig. 6).

The two validation-only studies externally validated commercially available models, and these were completely independent. The remaining models were evaluated by splitting the pre-processed dataset into training and test sets. Split-sample internal validation-only, or split-sample with leave-one-out or grid search cross-validation were most common. The predictive performance of the accelerometer-based models was described using classification accuracy in all models, and this was the only measure of performance in three models. One model’s performance was assessed using measures derived from confusion matrices [38]. Other measures (Cohen’s kappa, repeated measures analysis of variance (ANOVA), and detection error) were used in two models [40, 41]. Overall, the accelerometer-based models reported good-to-moderate classification accuracies.

#### Other Models

In addition to addressing the exercise classification problem, three studies reported models to predict the number of repetitions completed [37, 40, 41], and one study reported a model for 1RM prediction [37]. Of those reporting repetition counting, the mean counting error varied between 0.008 and 0.25 per repetition.

### Other Technologies

**Fig. 8:**
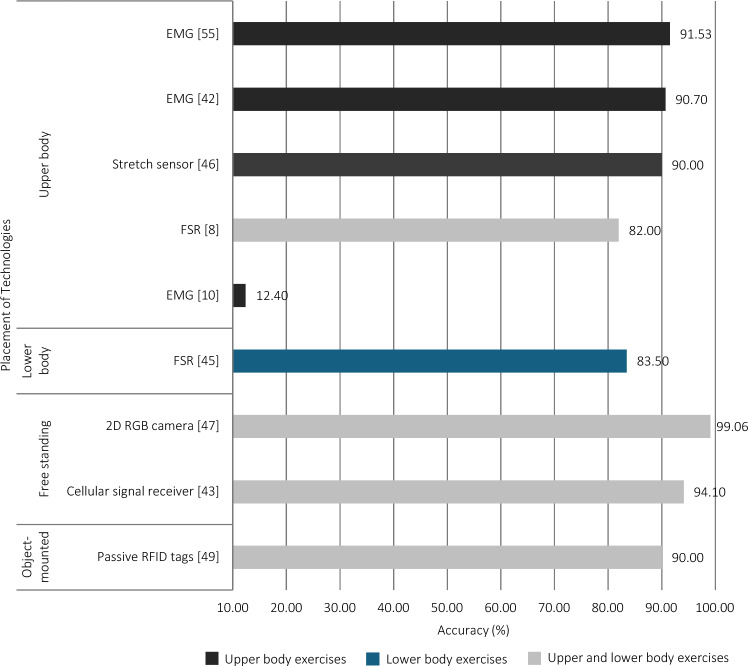
Overview of reported accuracy measures for other technologies according to the placement of technologies and exercising body segment. *EMG* electromyography, *FSR* force-sensitive resistor, *RGB* red, green, blue, *RFID* radio frequency identification, *2D* two-dimensional

Other technologies included electromyography (EMG) (*n* = 3); force-sensitive resistors (FSRs) (*n* = 2); two-dimensional (2D) red, green, blue (RGB) camera (*n* = 1); stretch sensor (*n* = 1); capacitive proximity sensor (*n* = 1); cellular signal receiver (*n* = 1); active sonar system (*n* = 1); and passive radio frequency identification (RFID) tags (*n* = 1) (Table 3). Owing to the small sample of studies that used these technologies, it is difficult to determine the methodology that may or may not have influenced model performance. However, some differences were noted. Most of the studies that used other technologies reported using custom-built devices, including a smart glove, textile sensor, sensing mat, and signal receiver. Devices were free standing, mounted to an object used during exercise, or were worn on the participant’s upper or lower body. When devices were affixed to the body segment with the most movement during the exercise or were not positioned on the participant, models appeared to perform better (Fig. 8). The models tended to classify small sets (3–13 exercises) of free-weight, machine-resisted, and/or bodyweight resistance training exercises. Two models classified a large set of 30 free-weight, machine-resisted, and bodyweight exercises [10, 55]. The algorithms employed across studies that used other technologies were diverse, with most using a different algorithm (Fig. 5). Of these, the SVM algorithm using input data obtained from a 2D RGB camera had the best performance among the studies that utilised other technologies (Fig. 6). The predictive performance of the models was described using classification accuracy in all but two of the models. Measures derived from confusion matrices were also commonly used to evaluate the models’ performance (6/10).

**Table 3:** Summary of studies that investigated other technologies for prediction of exercise during resistance training (*n* = 11)

Technology	Study	Exercise	Predictors	Prediction model algorithm	Validation method	Performance variables	Findings
Electromyography (EMG)	Chun et al. [42]	Wrist curl, biceps curl, dumbbell kickback	Normalised EMG data	Decision tree (DT)	Train and test with validation split	Training accuracy, test accuracy, sensitivity, false negative rate	Train ACC: 91.6; test ACC: 90.7; TPR: 91.23; FNR: 8.77
	Koskimäki and Siirtola [10]	Close-grip bench press, skull crusher, triceps pushdown, triceps dips, overhead triceps extension, triceps dumbbell kickback, spider curl, dumbbell alternating biceps curl, incline hammer curl, concentration curl, cable curl, hammer curl, upright row, lateral raise, front raise, shoulder press, car drivers, lying rear delt raise, bench press, incline dumbbell fly, incline dumbbell press, dumbbell fly, push-up, leverage chest press, seated cable row, one-arm dumbbell row, behind-neck lat pulldown, barbell row, reverse grip, reverse-grip barbell row, lat pulldown	Standard deviation, mean, minimum, maximum, median, percentiles, sum of data value over 25, 50, 100, 150 and 200	Unclear; linear discriminant analysis (LDA) and/or quadratic discriminant analysis (QDA)	Train and test with leave-one-out cross validation	Accuracy	ACC: 12.4
	Mekruksavanich and Jitpattanakul [55]	Seated cable rows, one-arm dumbbell row, wide-grip pulldown behind the neck, bent-over barbell row, reverse-grip bent-over row, wide-grip front pulldown, bench press, inclined dumbbell flyers, inclined dumbbell press, dumbbell flyers, push-ups, leveraged chest press, closed-grip barbell bench press, bar skull crusher, triceps pushdown, bench dip/dip, overhead triceps extension, triceps dumbbell kickback, spider curl, dumbbell alternate biceps curl, inclined hammer curl, concentration curl, cable curl, hammer curl, upright barbell row, side lateral raise, front dumbbell raise, seated dumbbell shoulder press, car drivers, lying rear-delt raise	NI	Combined methods; convolution neural network, residual connections, and bidirectional gated recurrent units (CNN-ResBiGRU)	Train and test with fivefold cross validation	Accuracy, F1-score, loss	ACC: 91.53; F1: 74.49; loss: 0.34
Cellular signal receiver	Teng et al. [43]	Squats, lunges, sit-ups, deadlifts, crunches, push-ups	Low-frequency features from Fast Fourier Transform and discrete wavelet transform	Cubic support vector machines (SVM)	Train and test with fivefold cross validation	Accuracy, classification permanence after 3 months	ACC: 94.1; permanence: 81%
Active sonar system	Fu et al.[44]	Push-up, sit-up, squat, segmental rotation, trunk rotation, superman, bridge, quadruped	Features from the transformed frequency time spectrum, spectrograms	Siamese few-shot learning	Train and test with fivefold cross validation	F1-score	F1: 94.11
Force-sensitive resistor (FSR)	Akpa et al. [8]	Bench dips, mountain climber, dumbbell curl, knee-pull-in, knee-twist-in, plank leg raises, triceps dips, push-up, side-to-side lunge, wall push-up	Mean, standard deviation, number of above mean crossing, number of below mean, number of peaks, skewness, kurtosis, band power, mean frequency, max power spectrum	Ensemble subspace k-nearest neighbour (k-NN)	Train and test with tenfold cross validation	Accuracy, F1-score	ACC: 82; F1: 83
	Zhou et al. [45]	Leg press, seated leg curl, leg extension, cross trainer	Magnitude range, average absolute of the 1st-order derivatives, standard deviation of the 1st-order derivatives, range of the 1st-order derivatives, central frequency of Fast Fourier Transform spectrum, mean magnitude of frequency band portions, count number of bins, local maxima, local minima, maxima-to-minima ratio, first 3 central moments, Hu’s 7 moments of the region of interest	Confidence-based adaptive boosting (ConfAdaBoost.M1)	Train and test with tenfold cross validation	Accuracy, F1-score	ACC: 83.5; F1: 85.2
Stretch sensor	Nguyen et al.[46]	Wrist curl, biceps curl, triceps extension, dumbbell kickback	Continuous time series data	One-dimensional convolution neural network (CNN)	Train and test with validation split	Training accuracy, test accuracy	F1: 93.77
Two-dimensional red, green, blue (RGB) camera	Cheng et al. [47]	Biceps curl, triceps extension, chest press, chest fly, shoulder press, seated row, deadlift, pulldown, dumbbell squat, moving hand squat, superman side squat, hands up, hand circle	Three-dimensional body key points, joint angles	Support vector machines (SVM)	Train and test with tenfold cross validation	Accuracy	ACC: 99.06
Capacitive proximity sensor	Fu et al. [48]	Push-up, sit-up, squat, segmental rotation, trunk rotation, superman, bridge, quadruped	Mean, variance, skewness, kurtosis, sample entropy, absolute sum of changes, autocorrelation, number of peaks, Fast Fourier Transform coefficients	Parallel branch early fusion (PBEF) convolution neural network (CNN)	Train and test with leave-one-out cross validation	F1-score	F1: 93.5
Passive radio frequency identification (RFID) tags	Ding et al. [49]	Concentration biceps curl, overhead triceps extension, flat bench biceps curl, alternating biceps curl, single arm bent-over row, single arm lateral raise, shoulder press, incline chest fly, front raise, squat	Doppler profiles	Decision tree (DT)	Unclear	Accuracy, precision, sensitivity, false positive rate	ACC: 90; PPV: 90; TPR: 91; FPR: 1.1

*ACC* accuracy, *PPV* positive predictive value, Precision; *TPR* true positive rate, Sensitivity; *FPR* false positive rate, *FNR* false negative rate

#### Other Models

In addition to addressing the exercise classification problem, five studies reported models to predict the number of repetitions completed [8, 43–45, 48], and three studies reported models for other performance metrics, such as repetition time [43, 49] and subjective difficulty [45]. Of those reporting repetition counting, the mean counting error varied between 0.069 and 0.29 per repetition.

### Combined Technologies

A combination of technologies was used in eight models. Of these, IMUs were used in five models in combination with EMG (*n* = 3), electrocardiogram (ECG) (*n* = 1) and RGB cameras (*n* = 1) (Table 4). When IMUs and EMG were used in combination, model performance in a single study was improved (55.80% and 12.40% versus 58.70%) [10]. However, the accuracy was notably lower than all other IMU- and EMG-based models (Fig. 6). Similarly, another study also showed improvements in model performance with IMU and EMG combined (96.96% and 91.53% versus 97.29%) [55]. However, the results were not substantially more accurate than studies that used IMUs alone. Radhakrishnan et al. [58] also combined IMUs and EMG and achieved an accuracy of 96.00%. As such, it is uncertain whether using both technologies would more broadly lead to improvements in model accuracy. Accelerometers were used in combination with ECG in one model [52]. Whilst the performance was not considerably different, the model that used the same algorithm but with only an accelerometer [38] appeared to have better accuracy than when combined with ECG data (82.00% versus 80.25%).

**Table 4:** Summary of studies that investigated combined technologies for prediction of exercise during resistance training (*n* = 8)

Technology	Study	Exercise	Predictors	Prediction model algorithm	Validation method	Performance variables	Findings
Inertial measurement unit (IMU) and electromyography (EMG)	Koskimäki and Siirtola [10]	Close-grip bench press, skull crusher, triceps pushdown, triceps dips, overhead triceps extension, triceps dumbbell kickback, spider curl, dumbbell alternating biceps curl, incline hammer curl, concentration curl, cable curl, hammer curl, upright row, lateral raise, front raise, shoulder press, car drivers, lying rear delt raise, bench press, incline dumbbell fly, incline dumbbell press, dumbbell fly, push-up, leverage chest press, seated cable row, one-arm dumbbell row, behind-neck lat pulldown, barbell row, reverse grip, reverse-grip barbell row, lat pulldown	Mean, standard deviation, minimum, maximum, median, percentiles, zero and mean crossing, Fast Fourier Transform sums, squared sums using all channels, sums of wavelet decompositions using different bookkeeping vectors, autocorrelation, cross-correlation, standard deviation, mean, minimum, maximum, median, percentiles, sum of data value over 25, 50, 100, 150 and 200	Unclear; linear discriminant analysis (LDA) and/or quadratic discriminant analysis (QDA)	Train and test with leave-one-out cross validation	Accuracy	ACC: 58.7
	Radhakrishnan et al. [58]	Correctly performed sit-up, incorrectly performed sit-up, correctly performed curl up, incorrectly performed curl up, correctly performed transverse abdominis with straight leg raise, incorrectly performed transverse abdominis with straight leg raise, correctly performed reverse curl up, incorrectly performed reverse curl up	NI	Convolutional neural network (CNN) with random under sampling boosted classifier	Train and test with fivefold cross validation	Accuracy	ACC: 96
Inertial measurement unit (IMU) and electromyography (EMG)	Mekruksavanich and Jitpattanakul [55]	Seated cable rows, one-arm dumbbell row, wide-grip pulldown behind the neck, bent-over barbell row, reverse-grip bent-over row, wide-grip front pulldown, bench press, inclined dumbbell flyers, inclined dumbbell press, dumbbell flyers, push-ups, leveraged chest press, closed-grip barbell bench press, bar skull crusher, triceps pushdown, bench dip/dip, overhead triceps extension, triceps dumbbell kickback, spider curl, dumbbell alternate biceps curl, inclined hammer curl, concentration curl, cable curl, hammer curl, upright barbell row, side lateral raise, front dumbbell raise, seated dumbbell shoulder press, car drivers, lying rear-delt raise	NI	Combined methods; a convolution neural network, residual connections, and bidirectional gated recurrent units (CNN-ResBiGRU)	Train and test with fivefold cross validation	Accuracy, F1-score, loss	ACC: 97.29; F1: 92.68; loss: 0.12
Inertial measurement unit (IMU) and electrocardiogram (ECG)	Qi et al. [50]	Bench press, squats, lunges, bent-over row, deadlift, good morning, shrugs, front raises, overhead extensions, biceps curls	Mean, standard deviation, covariance, variance, min, max, correlation, root mean square, signal magnitude vector, Fast Fourier Transform energy, entropy, R wave	Combined methods; one-class support vector machine (OC-SVM) and learning vector quantization hidden Markov model (LVG-HMM)	Train and test tenfold cross validation	No overall performance measures	
Inertial measurement unit (IMU) and red, green, blue (RGB) camera	Hwang et al. [51]	Chest press, shoulder press, seated row, biceps curl, overhead triceps extension	Quaternion (xq, yq, zq, wq), gyroscope (xg, yg, zg), and acceleration (xa, ya, za) data of the IMU sensor and estimated joint positions (*x*- and *y*-coordinates) of 25 major joints	Convolution neural network (CNN)	Train and test only	Accuracy	ACC: 98.83
Accelerometer and electrocardiogram (ECG)	Hussain et al. [52]	Butterfly, cable crossover, chest press, decline press, dumbbell fly, incline press, push-ups, barbell biceps curl, triceps dips, dumbbell biceps curl, dumbbell extension, preacher curl, cable extension, high pulley curl, 4 × 4 dumbbell, front raise, back shoulder press, upright row, front shoulder press, lateral raise, shrugs, pull-ups, front pulldown, lower pulley, vertical traction, back pulldown, one-arm dumbbell row, T-bar row, squats, leg press, leg extension, leg curl, abductor, adductor, lunge, leg raises, crunches, cross bicycles, plank, mountain climber, leg scissor, boat	NI	Bidirectional long short-term memory (LSTM)	Train and test only	Accuracy, loss	ACC: 80.25; loss: 1.3
Electromyography (EMG) and red, green, blue (RGB) camera	Yoo et al. [53]	Biceps curl, dumbbell kickback	Image contained features that were fair to be recognised by human eyes produced by manipulations of mth channel sEMG data and joint angle data	Convolution neural network (CNN)	Train and test with tenfold cross validation	Test accuracy, training loss	ACC: 99.7; loss: 1.82
Rotary encoder and load cell	Gou and Wang [54]	Unclear; leg press, squat and/or incline bench press	Acceleration, velocity, cable force, displacement, power, weightlifting duration, maximum time, minimum time, ranges, fluctuations, amplitudes, inclines, relations, and declines features	Probabilistic neural network (PNN)	Train and test with validation split	Accuracy, precision, sensitivity, F1-score	ACC: 98.4; PPV: 99.42; TPR: 98.54; F1: 98.24

*NI* no information reported by the study, *ACC* accuracy, *PPV* positive predictive value, Precision; *TPR* true positive rate, Sensitivity

When IMU data were combined with vision data obtained from an RGB camera, model performance appeared to improve [51]. A similar result was found when an RGB camera was used in combination with EMG [53]. Model performance appeared much better when EMG data were combined with vision data obtained from an RGB camera (99.70% [53] versus 91.53% [55]). A rotary encoder and load cell were combined in one model [54]. These technologies and the algorithm used to develop the model (i.e. probabilistic neural networks) were not used in any other study. Thus, it is unclear whether the measurement technology, predictive modelling approach or a combination were responsible for the reported high accuracy (98.40% [54]).

#### Other Models

In addition to addressing the exercise classification problem, two studies reported models to predict the number of repetitions completed [50, 51], and two studies reported models for other performance metrics, such as 1RM prediction [51] and training zone prediction [52]. Of those reporting repetition counting, the mean counting error varied between 0.061 and 0.379 per repetition.

## Discussion

The aim of this systematic review was to establish the current level of evidence for the technological approaches to classifying resistance training exercises and summarise and compare their performance. A total of 49 models from 44 studies were included, using primarily IMUs and to a lesser extent stand-alone accelerometers. Whilst wrist-worn IMUs offer excellent accuracy, even for classifying lower body exercises, marginal improvements in model performance above this were observed for devices measuring both the upper and lower body, particularly when capturing exercise data for movements of the upper and lower segments of the body. The nature of the classification problem (i.e. the number of exercises and how discernible the exercises were) also had a notable impact on model performance. This indicates that sensor placements and the selection of exercises are important considerations when developing technology that aims to classify resistance training. Overall, accurate exercise classification is possible with sensor-based technology, although the end-user availability of such technology is limited.

The use of multiple devices in some studies did not appear to improve model performance. Of note, when devices measured movement of both the upper and lower body, for example, when IMUs were placed on both the upper and lower body, prediction models seemed to perform slightly better. In addition, when devices were positioned on the segment of the body with the greatest movement during the exercise, classification accuracy appeared to improve. This is likely because sensor readings can vary substantially for the same exercise across different placement sites [59]. That said, devices, and in particular IMUs, placed on the wrist show excellent accuracy (> 90%), even for classifying exercises of the lower body, and this offers a practical and convenient solution to automatically identify the exercise being performed. Additionally, other measurement technologies worn by the athlete, such as EMG or smart materials, are also very accurate at predicting the exercise that is being completed (~ 90%). External devices, placed near the athlete, such as on a dumbbell or weight stack, perform well (accuracy ~ 95%) but only track a small number of exercises (< 12) and are practically less convenient. Given the strong performance of IMUs placed on the wrist, the practical benefits of alternative approaches are unclear. Furthermore, data sampling rates consistent with that seen in commercially available smart watches appear to perform as well as more expensive solutions. Thus, app-based approaches for currently available devices may offer a feasible and accurate approach to tracking resistance training exercise.

Inertial measurement units and stand-alone accelerometers were the most used technologies, with IMUs generally performing best. Most studies achieved favourable results for the prediction of resistance training exercises, with several studies achieving > 90% classification accuracy (*n* = 22). It is interesting to note that only three studies involved > 20 exercises, and more than half involved < 10 exercises. In prediction modelling, classification accuracy can be influenced by the number of outcomes. This is clearly shown in the accelerometer studies, where model performance tended to decrease when more exercises were included in the classification problem. Aligned with this, the discernability of outcomes can also significantly affect the accuracy of predictions. Exercises that can be easily differentiated from one another generally lead to a higher classification accuracy. This is because the model learns to recognise patterns that differentiate one outcome from another [60], which is easier when these exercises are not similar. An example of this can be observed in Steeves et al. [41] when similar exercises were included (i.e. shoulder press and bench press) and the model had trouble differentiating them and they were regularly predicted as each other (32% of the time). Thus, when aiming to develop and validate technology that can accurately categorise different exercises, it is important to include a range of exercises that have similar movement patterns and ranges of motion and are performed in the same plane of motion [61].

The algorithms used to build and train predictive models were diverse, with CNN and SVM being the most common algorithms for model development. Algorithms that produced some of the most accurate predictions were also used in studies reporting some of the poorest predictions. Only the model with the best performance from each of the included studies was selected for inclusion in this review. It is notable that in some studies where numerous algorithms were tested, the best performing algorithm differed. For example, Balkhi and Moallem [26] and Guo et al. [32] compared the same algorithms but identified RF and SVM, respectively, as producing the best predictions. With such inconsistent results, it is still unclear whether certain algorithms outperform others in terms of producing accurate predictions, or whether other factors, such as the placement of devices and the nature of the classification problem, are greater determinants of model performance. Thus, researchers and developers should evaluate the performance of multiple modelling approaches to ensure that they can achieve the most accurate approach to exercise prediction with their chosen technology, population and exercises.

One particularly interesting consideration is that only six of the studies available were on consumer-level technology [7, 28, 34, 37, 41, 43]. However, two of these are no longer available. Furthermore, three of these studies required some level of programming knowledge, which likely mitigates their practical use. The lack of technology that is available to the consumer also points to available technology that has not yet been scientifically validated for the classification of exercises. That said, the broader findings of this review suggest that consumer-level devices are likely capable of exercise classification. Whilst our review was primarily focused on exercise detection, other metrics such as repetition counting, 1RM prediction, exercise ‘form’ and other performance metrics related to exercise are relevant, as they will most likely be dependent on the correct classification of the exercise. Several studies reported models to predict the number of repetitions completed (*n* = 19). These show promising results, with the mean counting error varying between 0.0 and 0.59.

Rigorous guidelines have been developed to encourage the execution and reporting of high-quality prediction modelling studies [62]. However, as evidenced by the uncertain or high risk of bias of the reviewed studies, research pertaining to exercise classification is yet to adopt these guidelines. This can be explained, to an extent, by the rapid development of available technology in this field over the last 10 years (note that the earliest included study was published in 2015), and, thus, the motivation for industry to solve this problem in a time-efficient manner. However, it is also worth noting that PROBAST places strict criteria that may not apply to modern machine learning approaches (e.g. EPV) or which are impractical outside of large public health datasets. Further, the PROBAST tool was originally designed for assessment of diagnostic and prognostics models of medical conditions, and elements of the tool are depended upon detailed reporting of model predictors and analysis [18]. In the context of the current research question, authors may elect not to disclose this information owing to its potential commercial value, leading to the reported high risk of bias across studies. That said, to ensure technology is robust to consumer expectations, it is important to systematically evaluate model performance. This includes an assessment of overall model performance, discrimination and calibration [62, 63]. Most of the included studies did not evaluate performance with such measures. Instead, only classification accuracy, or other measures derived from confusion matrices (i.e. sensitivity, specificity, precision and F1-score), was assessed.

### Standardising the Classification Problem

With classification problems being defined in different ways, direct comparisons between the performance of different technologies, sensor placements and predictive modelling approaches are not possible, and this may slow progress in this field. Thus, if the aim of a study is to classify a range of resistance training exercises, then we suggest standardising the classification problem, which may assist in advancing progress towards a solution. However, this must be balanced against the need for researchers and developers to rapidly respond to technological developments and industry demand. Therefore, we propose a three-phased approach to evaluating model performance that is sufficiently agile in phase 1, sufficiently rigorous and standardised in phase 2, thereby enabling comparisons between approaches, and bespoke in phase 3 to encourage the development of solutions beyond the standard problem. This three-phased approach can be found in Table 5, with researchers strongly recommended to use this approach so that rigorous and comparable outcomes can be achieved. In addition, consumers can make informed objective choices about the resistance training exercise tracking tools they select.

**Table 5:** Three-phase approach for evaluating model performance

	Phase 1: Piloting	Phase 2: The general resistance training problem	Phase 3: Specific sub-populations
Sample	• Small convenience samples may be used in the initial development stages. Investigators should be aware of the potential overfitting and optimism in model performance with small samples, and account for this in the latter stages	• A large sample of trained participants (i.e. at least 6 months of training experience, frequenting > 2 sessions a week) should be used	• Investigators may choose to sample specific sub-populations
Classification problem	• Investigators may choose to select upper OR lower limb-specific exercises • A standardised selection of exercises should be made, i.e. squat, deadlift, lunge, and calf raise (lower body) OR biceps curl, lateral raise, shoulder press, and bent-over row (upper body) • Investigators are encouraged to utilise a training protocol, including a standardised loading scheme	• Investigators should select upper AND lower limb-specific exercises. Variations of exercise are also recommended (e.g. flat bench press and incline bench press) • Studies should report exercise definitions, including the mode of exercise and any instructions on exercise performance • Investigators are encouraged to utilise a training protocol, including a standardised loading scheme • Between set rest periods (i.e. non-exercise time) should be included in the classification problem	• Investigators should select exercises specific to the sampled sub-population • Studies should report exercise definitions, including the mode of exercise and any instructions on exercise performance • Investigators are encouraged to utilise a training protocol (if relevant) • Between set rest periods (i.e. non-exercise time) should be included in the classification problem (if relevant)
Model evaluation	• Studies should always include an appropriate form of internal validation, such as bootstrapping or cross-validation. Split-sample validation may be used, but investigators should be aware of the potential risk of bias of such an approach	• Studies should always include an appropriate form of internal validation, such as bootstrapping or cross-validation	• Studies should always include an appropriate form of internal validation, such as bootstrapping or cross-validation
Model performance	• Model performance may be assessed with classification measures, including classification accuracy, sensitivity, specificity, or predictive values	• Model performance should be comprehensively assessed with measures of discrimination, calibration, and classification	• Model performance should be comprehensively assessed with measures of discrimination, calibration, and classification

## Conclusions

**Fig. 9:**
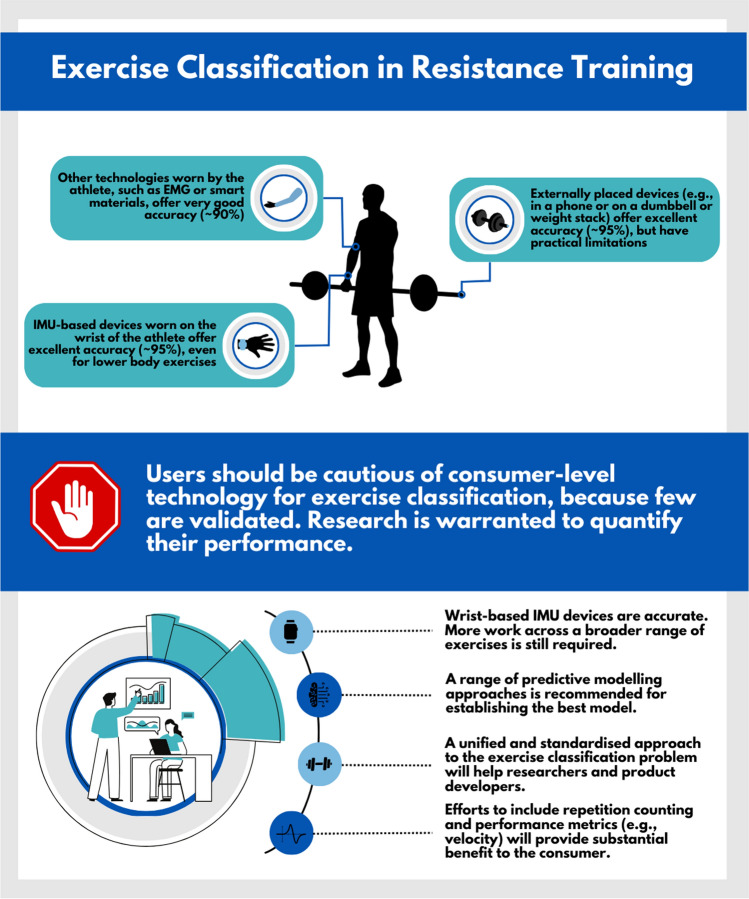
Infographic of review findings and implications for practitioners and researchers. *EMG* electromyography, *IMU* inertial measurement unit

This review highlights the various approaches that have been taken to develop predictive models that classify resistance training exercises. IMUs appear to be the most accurate and, when worn on the wrist of the athlete, offer excellent accuracy, even for lower body exercises. Other measurement technology worn by the athlete, such as electromyography and smart materials, also offers very good accuracy. Finally, externally placed devices, whilst offering excellent accuracy, have practical limitations that may compromise their feasibility (Fig. 9). Of note, the exercises included in the classification problem, and specifically how similar the exercises were, had a significant impact on model performance. Standardising this classification problem is strongly recommended as it will likely facilitate a clearer understanding of the best approach and inform consumers and future research in this area. Furthermore, ensuring technologies are robust to the prediction of a large range of exercises with similar movement patterns remains a priority and potential barrier to feasibility. Overall, accurate exercise classification is possible with sensor-based technology, although the end-user availability of such technology is limited. It is strongly advised that users be cautious of consumer-level technology, because few are scientifically validated.

## Supplementary Information

Below is the link to the electronic supplementary material.

Supplementary file1 (PDF 267 KB)

Supplementary file2 (XLSX 298 KB)

## Declarations

### Availability of Data and Materials

All data and material reported in this systematic review are from peer-reviewed journals. Data supporting the results reported in the review can be found in the Supplementary Material.
